# Editorial on Special Issue “Immunotherapy, Tumor Microenvironment and Survival Signaling”

**DOI:** 10.3390/cancers14010091

**Published:** 2021-12-24

**Authors:** Vita Golubovskaya

**Affiliations:** Promab Biotechnologies, 2600 Hilltop Drive, Richmond, CA 94806, USA; vita.gol@promab.com

## 1. Novel Immunotherapies

Recently, novel types of immunotherapies such as CAR-T cell therapy demonstrated efficacy in leukemia, lymphoma, and multiple myeloma [1,2,3]. CD19 and BCMA-CAR-T cell therapies were approved by FDA to treat patients with the above diseases. There are still several challenges for CAR-T cell therapy, including safe and effective antigen targets for solid tumors, overcoming a suppressive tumor microenvironment, and loss of antigen expression, among others [4,5].

One of the approaches to overcome potential CD19 loss during treatment with CD19 CAR-T cells was presented in a paper with novel CD37 and CD37-CD19-CAR-T cells [6]. The authors generated a novel CD37 antibody and engineered both novel Cd37-CAR-T cells and bispecific CD37-CD19-CAR-T cells that effectively targeted CD19+CD37+ lymphoma in vitro and in vivo [6].

Another group presented novel DCLK1 (doublecortin-like kinase 1) antibody and DCLK-1-CAR-T cells targeting colorectal cancers [7]. The authors demonstrated that DCLK1 was a marker of tuft cells (TC) and cancer stem cells (CSCs) and suggested that DCLK1-positive TCs participated in the initiation and progression of inflammation-associated cancer [8].

In another report, the authors reviewed different approaches to generate metabolically fit CAR-T cells to overcome the immune-suppressive tumor microenvironment [9]. Since it is known that metabolic pathways can control T cell proliferation, expansion, differentiation, and function, the authors describe different enzymes or protein regulators of metabolism that can be modulated to increase the efficacy of CAR-T cells [9]. One of the approaches is to induce expression of PPAR-gamma coactivator 1-α (PGC1-α), which controls mitochondrial biogenesis and results in an increased efficacy of T cells. Another approach is to generate CAR-T cells which secrete the antioxidant enzyme catalase to overcome the hypoxic tumor microenvironment [9]. Several other approaches are highlighted with CAR-T cells engineered to effect glycolysis, glutaminolysis, and the pentose phosphate pathway.

In an original approach to overcome potential antigen loss during immunotherapy, the authors engineered T cells with anti gp100 TCR antigen targeting PMEL (premelanosome protein) and anti-CSPG4 CAR targeting chondroitin sulfate proteoglycan 4 (also known as melanoma-associated chondroitin sulfate proteoglycan, MCSP, or high molecular weight melanoma-associated antigen, HMW-MAA) [10]. These armed T cells were generated with stable lentiviral delivery of gp100 TCR and transient delivery of CSPG4-CAR using RNA electroporation [10]. This represents a novel approach in immunotherapy which needs further optimization to be used in clinics.

## 2. Novel Approaches Targeting the Tumor Microenvironment

It is well known that the solid tumor microenvironment is hypoxic with high level of TGF-β which represses immunotherapy. Hypoxia is present in the case of solid tumors and in bone marrow niche where B cells reside [11]. One report studied the effect of hypoxia on function of CAR-T cells and found that hypoxia impaired CAR-T cell expansion and affected differentiation and cytokine production [12].

Most tumors are surrounded and infiltrated by TAM (tumor associated macrophages) that promote tumor motility, angiogenesis, metastasis, repress T cell functions, and inhibit the effect of chemo- or other immunotherapies [13]. The authors delivered special lytic proapoptotic peptides to block TAMs, eliminate circulating monocytes and macrophages [13].

One of the approaches to block tumors and tumor microenvironment players is to use antibodies to block programmed cell death 1 (PD-1) or its ligand 1 (PD-L1). One of the reports demonstrated that sequential use of PD-1 and PD-L1 antibodies can cause cardiotoxicity in patient with brain metastatic lung adenocarcinoma [14]. Thus, the authors conclude that the combinatory use of PD-1 and PD-L1 blockade, either sequentially or concurrently, should be used carefully to avoid cardiotoxicity [14].

Another report reviewed different agents to improve checkpoint inhibitor efficacy in clinic [15]. The authors report that while in melanoma and non-small cell lung cancer using immune checkpoint inhibitors results in a high efficacy, the response rate in other tumors, such as gastrointestinal cancers, breast cancer, sarcomas, and some genitourinary cancers remains low [15]. Several strategies are discussed that can improve efficacy such as use of predictive factors of the response (for example PD-L1 expression, tumor mutational burden, and clinical factors), combination therapy approach, use in addition to abscopal effect of radiotherapy other drugs such as microbiota modulators, anti-angiogenic agents, small molecules, and oncolytic viruses (drugs targeting co-inhibitory receptors) [15].

Another review focuses on the role of microRNA in the modulation of damage-associated molecular patterns (DAMP) [16]. Immunogenic cell death (ICD) which is triggered by several ICD-inducers released into tumor microenvironment plays a major role in stimulating anti-tumor response [16]. The exposure of DAMP such as calreticulin, ATP, Hsps and HMGB1 confers adjuvanticity to cancer cell death [16]. The authors review the main microRNA that target DAMPs such as Hsp 70, Hsp 90, HMGB1, calreticulin [16].

Another report summarizes interactions and crosstalk between myeloid-derived suppressor cells and regulatory T cells within the immunosuppressive tumor microenvironment [17]. The authors review role of beta-integrins, metabolic pathways, and cell-cell interactions as modulators of this cross-talk [17].

Another review focused on nanoparticles in cancer immunotherapy as the delivery agent of immunotherapeutic agents and as the immunomodulators [18]. The authors discussed nano-immunotherapy, targeting microenvironment with different nanoparticle-based agents, and overviewed future directions and challenges of this novel promising field [18].

Another review presented new mechanism of intercellular mitochondria transfer in both solid and hematological cancers [19]. The mitochondria transfer can change metabolic pathways and affect microenvironment and drug resistance mechanisms in different types of cancers.

## 3. Targeting Tumors and Tumor Microenvironment in Different Types of Cancer

Several reviews concentrated on targeting tumors and interplay with tumor microenvironment in different types of cancer such as triple negative breast cancer [20], breast cancer [21], pediatric cancer [22], and colorectal cancer [23,24].

The challenges in treating triple negative breast cancer patients with immune checkpoint inhibitors (ICI) are partly attributed to dysregulated angiogenesis, resulting in hypoxic tumor microenvironment, increased production of VEGF, EGF, and PDGF, thereby stimulating angiogenesis and metastasis [20]. Other challenges are long non-coding RNAs and microsatellite instability that cause immunosuppression and affect the efficacy of ICI [20]. Since there is limited T-cell infiltration in most breast cancers, the development of novel strategies to enhance sufficient lymphocyte infiltration, as well as to generate de novo T-cell responses that overcome the immunosuppressive tumor environment, may be the key to the success of this kind of therapy in breast cancer patients [21].

The role of dendritic cells is reviewed in immunotherapy of colorectal cancer [23]. New strategies to combine DC vaccination with check-point inhibitors open perspectives for a more effective treatment of disease [23].

The role of autophagy in the regulation of the tumor microenvironment for colorectal cancer, the specific mechanism by which autophagy is implicated in immune responses during CRC tumorigenesis, and the context of anticancer therapy is reviewed in [24].

## 4. Conclusions

This issue demonstrated the complex interaction between tumors and tumor microenvironment, reviewed cross talk and interplay between them, and provided the best strategies for overcoming challenges in targeting tumors with repressive immune environment. The reports provided future directions in increasing anticancer therapies.
